# Petrogenesis of extra-large flake graphite at the Bissett Creek deposit, Canada

**DOI:** 10.1007/s00126-022-01145-9

**Published:** 2022-12-05

**Authors:** Cameron Drever, Carson Kinney, Chris Yakymchuk

**Affiliations:** Department of Earth and Environmental Sciences, University of Waterloo, Waterloo, Canada

**Keywords:** Graphite, Flake, Metamorphism, Stable Isotope, Thermodynamic Modeling

## Abstract

The petrogenesis of extra-large flake graphite is enigmatic. The Bissett Creek graphite deposit, consisting of flake graphite hosted in upper-amphibolite facies quartzofeldspathic gneisses and rare aluminous gneisses, provides an analogue for graphite exploration. In the Bissett Creek gneisses, graphite is homogeneously distributed and composes 2–10 vol. % of the rocks. Disseminated graphite flakes (~ 1 to 6 mm in size) are interleaved with biotite and are petrologically associated with upper-amphibolite facies metamorphic mineral assemblages. Thermobarometry and phase equilibrium modeling yield peak temperatures of > 760 °C at 0.5–0.9 GPa. Whole-rock samples with abundant graphite yield δ^13^C_VPDB_ from − 28 to − 14‰. δ^34^S_VCDT_ values of sulfide-bearing samples vary from 10 to 15‰. Sulfur and carbon isotope values are compatible with a biogenic origin, flake graphite probably formed from metamorphism of in situ organic material. However, the variability of δ^13^C values from the deposit along with graphite microstructures suggest that carbon-bearing metamorphic fluid (or melt) generated during metamorphism may have remobilized carbon resulting in anomalously large to extra-large flake sizes. This may be a common mechanism globally to explain large graphite flake sizes where graphite formed through in situ metamorphism of organic matter is coarsened due to remobilization of CO_2_-rich fluids (or melt) during high-temperature metamorphism.

**Supplementary Information:**

The online version contains supplementary material available at 10.1007/s00126-022-01145-9.

## Introduction

Flake graphite is a crucial component in advanced and green energy technologies that rely on Li-ion batteries (Olson 2016) and is classified as a critical mineral (European Commission 2020; Natural Resources Canada 2021). Larger grain sizes and scarce inclusions in graphite are optimal for integration as anodes into such batteries, and large flake sizes have a higher thermal stability that is important for refractory applications (Taylor 2006). Flake graphite is generally mined from metasedimentary rocks in high-temperature metamorphic terranes (Landis 1971; Soman et al. 1986). Flake graphite is generally thought to form through in situ metamorphism of organic material; some of this organic material will become graphite, and some may break down into carbon-bearing fluids that can mobilize (Buseck and Beyssac 2014; Simandl et al. 2015). By contrast, vein (also known as lump) graphite precipitates from carbonic fluid and may have large crystal sizes (Luque et al. 2012), but these deposits are usually less common. Although graphite crystallinity and purity are also important, graphite with larger grain sizes is generally more valuable (Mitchell 1993). The largest classification of graphite, extra-large or jumbo (+ 50 mesh, or > 300 μm), is the most valuable category of flake graphite.

The two end-member genetic models for flake graphite mineralization are graphitization (conversion of organic carbon to graphite) and hydrothermal deposition (Luque et al. 2012; Beyssac and Rumble 2014); however, some graphite deposits are formed through a combination of both (Papineau et al. 2010; Luque et al. 2012; Parnell et al. 2021b). Notwithstanding the uncertainty regarding the formation of extra-large flake sizes, protolith clay content and the development of granoblastic microstructures (Scherba et al. 2018) may play critical roles. However, it is unclear if extra-large flake graphite deposits may also be influenced by hydrothermal carbon mobilization in addition to in situ metamorphic processes.

In this contribution, we combine fieldwork, petrography, carbon isotope analysis of graphite, sulfur isotope analysis of sulfide minerals, thermobarometry, and phase equilibrium modeling to determine the petrogenesis of the Bissett Creek graphite deposit in the Proterozoic Grenville Province in Canada. Although graphite purity and crystal size are important economic considerations, the Bissett Creek deposit is notable for preservation of coarse flakes after beneficiation (Leduc 2013). We test the hypothesis that the graphite has a biogenic origin and that it was generated at high-temperature metamorphic conditions and discuss the possibility of subsequent carbon mobility and graphite coarsening. Finally, we explore the general implications for the prospectivity of large- to extra-large flake graphite in other high-temperature metamorphic terranes.

## Regional geology

### Grenville Orogen

**Fig. 1:**
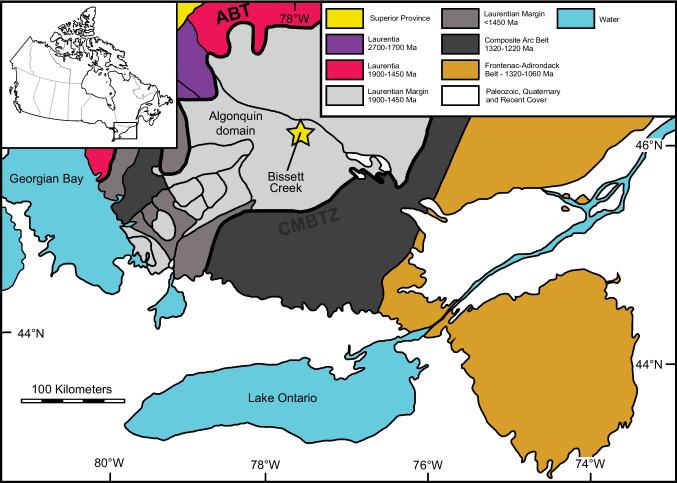
Geology of the southwestern Grenville Province. ABT: Allochthon Boundary Thrust. CMBTZ: Central Metasedimentary belt boundary tectonic zone. Modified from Carr et al. (2000)

The Bissett Creek graphite deposit is located in the Proterozoic Algonquin domain, which is a crustal block of the Grenville Province (Fig. 1). The Algonquin domain formed due to the accretion of 1800–1690 Ma arc-related rocks to the margin of the Archean Laurentian craton (Carr et al. 2000). After accretion, the pre-Grenvillian Laurentian Margin underwent several episodes of deformation and metamorphism, including (1) > 1600 Ma events from pre-Grenvillian periods of metamorphism and deformation, (2) 1450–1420 Ma events associated with magmatic activity, and (3) 1120–980 Ma compressional and extensional tectonism related to the Grenville Orogen (Carr et al. 2000; Rivers et al. 2012); this last episode is the dominant high-temperature metamorphic event that affected rocks in the southeastern Grenville Province.

The Grenville Province in Ontario is composed of three lithotectonic domains that each have a distinct tectonic history. From southeast to northwest, these include the Frontenac–Adirondack Belt, the Composite Arc Belt, and pre-Grenvillian Laurentia and its margin (Carr et al. 2000). Pre-Grenvillian Laurentia and its margin (previously known as the Central Gneiss Belt (Carr et al. 2000)) are bounded to the west by the Grenville Front Tectonic Zone (GFTZ) and to the east by the Central Metasedimentary Belt boundary thrust zone (Fig. 1). Both boundaries are major shear zones that were active during Grenville orogenesis (Davidson et al. 1985). In the southeastern Grenville Province, pre-Grenvillian Laurentia is made up of gently dipping, upper-amphibolite to granulite-facies orthogneiss with minor supracrustal rocks (Ketchum and Davidson 2000). Pre-Grenvillian Laurentia has been subdivided into several thrust sheets that represent different structural levels with distinct tectonometamorphic histories; individual sheets are bound by high strain zones (Culshaw et al. 1983; Davidson 1984; Rivers et al. 1989).

The Algonquin domain represents the lowest structural level of Laurentian Margin rocks (Ketchum and Davidson 2000) and it is underlain by rocks of the pre-Grenvillian Laurentian Craton and overlain by migmatitic gneisses of the Muskoka Domain (Carr et al. 2000). The western margin of the lower thrust sheet is known as the allochthon boundary thrust (Ketchum and Davidson 2000). Two major episodes of magmatism affected the region from 1.8 to 1.6 Ga and from 1.5 to 1.4 Ga (Carr et al. 2000; Ketchum and Davidson 2000). The Algonquin domain is mostly composed of quartzofeldspathic gneisses with rare paragneisses and metamafic rocks; most units record a complex polyphase deformation history.

### Geology of the Bissett Creek deposit

The geological observations presented here are based on outcrop and drill core samples, which build on previous technical reports of the deposit (Gignac et al. 2012; Leduc 2013) and the study of Taner et al. (2017). The Bissett Creek graphite hosts ~ 1.2 Mt of graphite (measured and indicated resources) with an average grade of 1.74 wt.% carbon (Leduc 2013). This grade is lower than many flake graphite deposits worldwide (e.g., Parnell et al. 2021a), but Bissett Creek is notable for its anomalously large flake size; > 70% of the flake graphite concentrate is classified as large- or extra-large flake (Leduc 2013).

**Fig. 2:**
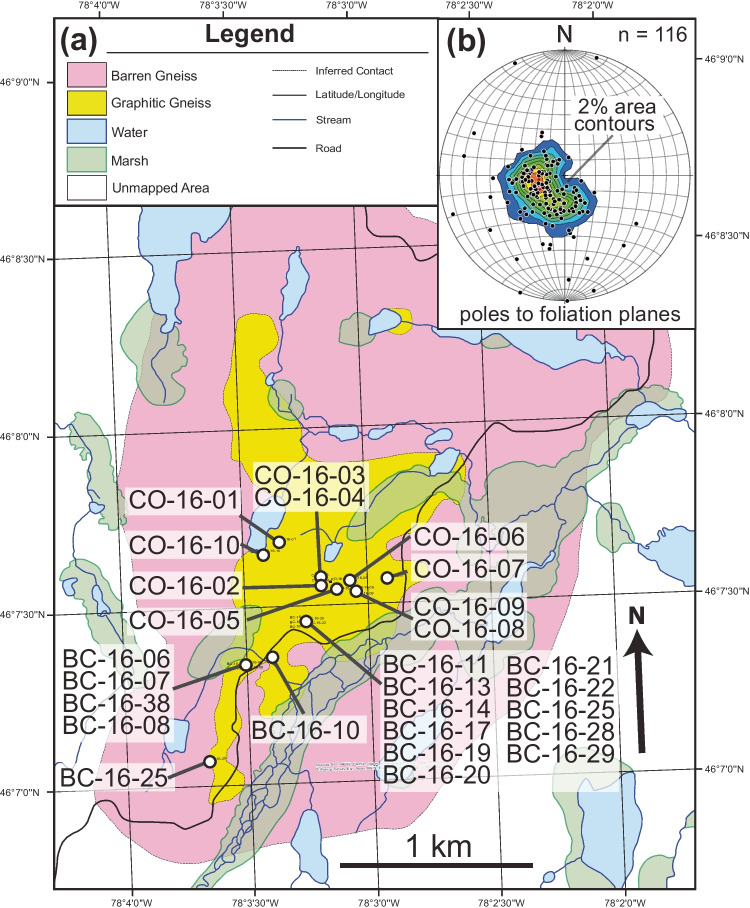
**a** Geological map of the Bissett Creek deposit. **b** Equal area stereonet of foliation measurements. Based on a geological map from Leduc (2013)

The geology of the Bissett Creek graphite deposit can be broadly divided into barren quartzofeldspathic gneisses and subordinate graphite-bearing gneisses (Fig. 2a). No (meta)carbonate layers were observed in outcrop or drill core. Considerable weathering, vegetation, and overburden hinder further subdivision of the graphitic and barren gneisses in outcrop; therefore, core samples were used to differentiate graphitic units. Contacts between the graphitic gneisses are gradational over a scale of centimeters to decimeters. The barren and graphitic gneisses generally strike southeast to northwest with gentle to moderate dips to the east, which is consistent with the regional foliation (Fig. 2b).

**Fig. 3:**
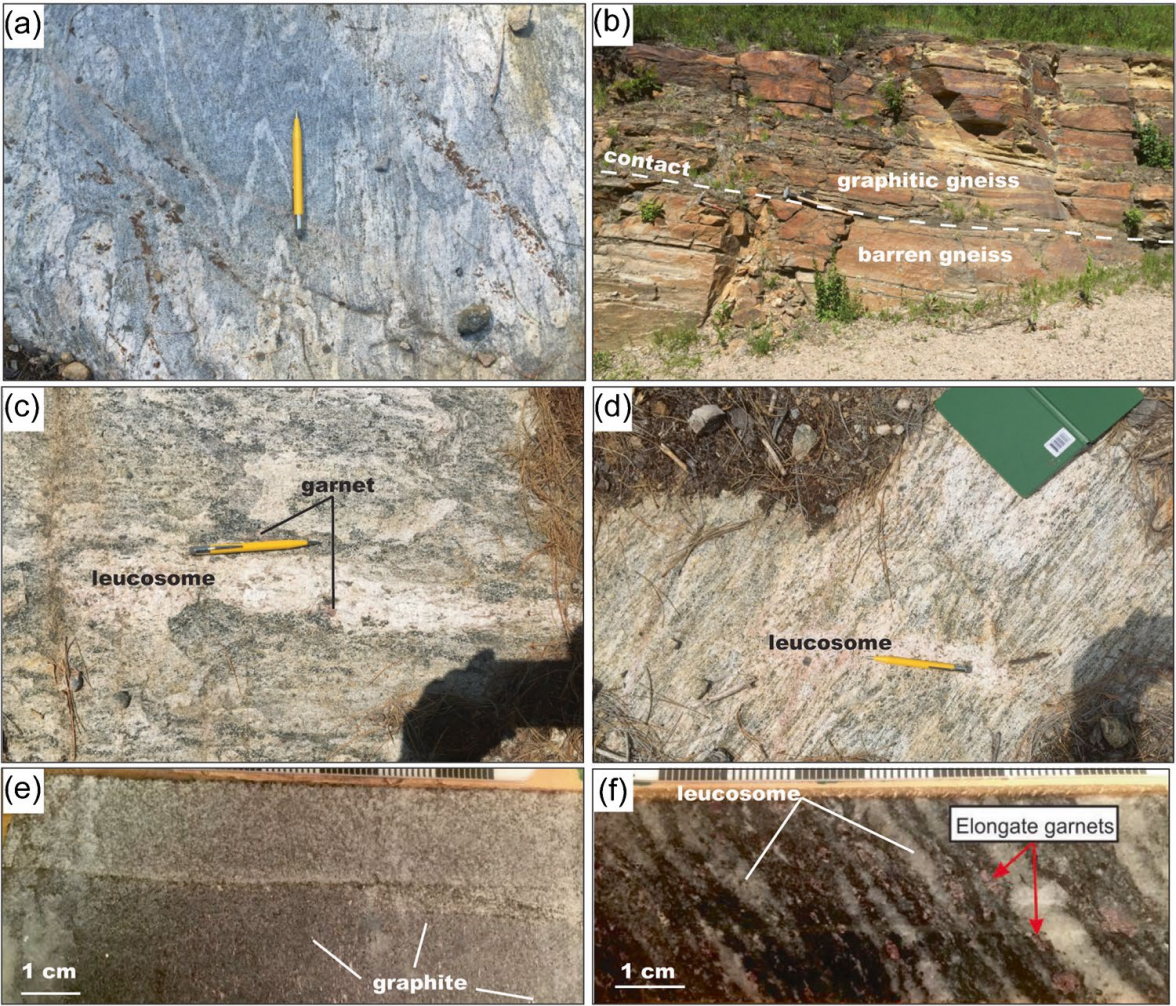
Representative field and core photos of select geological units at Bissett Creek. **a** Isoclinal folding in barren gneiss. **b** Foliation-parallel contact between the barren and graphite-bearing gneiss. **c** Folded leucosome with garnet indicative of in situ partial melting. **d** Pucker structure showing the bending of the foliation into the leucosome indicative of volume loss associated with melt extraction. **e** Disseminated graphite in the graphite-bearing quartzofeldspathic gneiss in core. **f** Elongate garnet and 20 vol.% leucosome in garnet–sillimanite gneiss in core

Graphitic gneisses are underlain and overlain by barren gneiss, which are both variably folded into isoclinal folds (Fig. 3a). The contact between the graphitic gneisses and the barren gneisses is sharp in outcrop (Fig. 3b) and in drill core. The barren gneisses contain several units based on metamorphic mineral assemblage that include biotite-rich quartzofeldspathic gneiss, amphibole-bearing quartzofeldspathic gneiss, and garnet–sillimanite gneiss. Some of the biotite-rich quartzofeldspathic gneisses contain leucosome with minor garnet (Fig. 3c), which is an indicator of in situ partial melting (e.g., Vernon 2011; Brown 2013; Yakymchuk 2021), and pucker structures that are suggestive of melt extraction (Fig. 3d).

The graphitic gneiss can be differentiated into several units based on metamorphic mineral assemblages that include quartzofeldspathic gneiss, clinopyroxene-quartzofeldspathic gneiss, garnet–hornblende gneiss, and garnet–sillimanite gneiss. The primary relationship between these units is not apparent due to pervasive transposition during deformation, and contacts are usually sub-parallel to the dominant foliation. The dominant graphite-bearing unit—the graphite quartzofeldspathic gneiss—contains homogeneously disseminated graphite (Fig. 3e). The garnet–sillimanite gneiss forms relatively narrow (< 1 m) layers and contains ~ 20 vol.% leucosome and elongate garnet (Fig. 3f). The preservation of high-temperature suprasolidus mineral assemblages (e.g., garnet and sillimanite) probably required local anatectic melt loss (e.g., White and Powell 2010), although macroscopic evidence of melt loss is not apparent in these rocks.

## Methods

### Analytical methods

Compositions of silicate minerals were determined for eight samples that include four garnet–sillimanite gneiss samples from core (BC-10–06, CO-16–05, CO-16–08, CO-16–10), three garnet-hornblende gneiss from outcrop (BC-16–29, BC-16–20, BC-16–18), and one quartzofeldspathic gneiss from outcrop (BC-16–28).

Mineral compositions were determined quantitatively for garnet, biotite, amphibole, plagioclase, potassium, and feldspar using a JEOL JXA-8530F field-emission electron microprobe at the Earth and Planetary Materials Analysis (EPMA) Lab at Western University (Canada). Garnet was analyzed using 1-μm spots along a line to assess compositional variation with an accelerating voltage of 15 kV and 20 nA current. Biotite, amphibole, plagioclase, and potassium feldspar were analyzed using 5-μm spots (two on each mineral, one on the rim and one in the core) with the same accelerating voltage and current. Acquisition time was ~ 3 min. Calibration of the instrument was undertaken prior to the start of analyses using a combination of natural and synthetic standards. The data was reduced using the built-in ZAF corrections in the JEOL software.

Whole-rock geochemistry of twenty-four samples (10 from drill core and 14 from outcrop) each weighing 1–2 kg was conducted at ActLabs (Ancaster, Canada). This included eight samples of quartzofeldspathic gneiss, eight samples of clinopyroxene gneiss, three samples of garnet–hornblende gneiss, and five samples of garnet-sillimanite gneiss. Major elements and trace elements were analyzed as part of the Actlabs 4 Lithoresearch package. Major element concentrations were determined by inductively coupled plasma optical emission spectrometer (ICP-OES), while trace element concentrations were determined using an inductively coupled plasma mass spectrometer (ICP-MS). Samples were dissolved using lithium metaborate and lithium tetraborate fusion. Molten material was dissolved in 5% nitric acid, and this solution was analyzed with ICP-OES and ICP-MS.

Total carbon and sulfur concentrations of 22 whole-rock powders were measured using Actlabs package 4F on an infrared (IR) detector. An accelerator material was combined with 0.2 g of sample material, placed in an induction furnace, and heated until carbon and sulfur combust. Combustion in a pure oxygen environment causes the formation of CO, CO_2_, and SO_2_. The CO is changed into CO_2_ in a catalytic heater assembly. Graphitic carbon of 19 whole-rock powders is measured on 0.5 g of sample material after multistage furnace treatment to remove all other forms of carbon prior to analysis, leaving only graphitic carbon. Afterwards, the same method as above was used to determine carbon contents.

Carbon isotope values were measured on the same 24 whole-rock powders as analyzed for lithogeochemistry at the Queen’s Facility for Isotope Research (QFIR). Samples (0.2–0.6 g) were weighted into tin capsules. Combustion was followed by analysis in a Costech ECS 4010 Elemental Analyzer coupled to a Thermo-Finnigan DeltaPlus XP Continuous-Flow Isotope Ratio Mass Spectrometer. The δ^13^C values obtained from the measurement are reported relative to the VPDB international scale (Coplen et al. 2006). Precision of δ^13^C values was 0.2‰.

Sulfur isotope ratios were measured on the same whole-rock powders as analyzed for lithogeochemistry at the Environmental Isotope Laboratory at the University of Waterloo. Samples ranging from 1.3 to 3.1 mg (depending on the concentration of sulfur) were loaded in 3.5 × 5 mm tin capsules. Loaded tin capsules are dropped into the Costech ECS 4010 Elemental Analyzer coupled to an Isochrom CF-IRMS. The isotopic composition of the sample is determined through combustion conversion (1000 ºC reactor, 90 ºC column, 115 ml/min He) of the solid sulfur-bearing materials into SO_2_ gas. The δ^34^S values are reported relative to the Vienna Canyon Diablo Troilite (VCDT; Krouse and Coplen 1997) international scale with a precision of 0.3‰.

### Thermobarometry

We conducted conventional garnet–biotite Fe–Mg exchange thermometry using the formulation of Holdaway (2000) for four samples of graphite-bearing sillimanite–garnet gneiss. We also use the Ti-in biotite thermometer of Henry et al. (2005) that was calibrated for biotite in metapelitic protoliths for graphite-bearing and graphite-absent mineral assemblages. As suggested by Henry et al. (2005), we use a 22 O normalization to calculate biotite formulae from electron microprobe analysis results. One caveat is that our rocks do not contain rutile and only rare ilmenite, which indicates that the calculated temperatures using the Ti-in-biotite thermometry probably underestimate peak temperature (Henry et al. 2005). We did not use graphite thermometry via Raman spectroscopy (e.g., Beyssac et al. 2002), given that graphite does not change crystallinity at > 650 °C; we expect much higher metamorphic temperatures than this limit of graphite thermometry based on previous regional *P–T* estimates (Anovitz and Essene 1990) and our own assessment.

### Phase equilibrium modeling

Phase equilibrium modeling is used to determine the P–T conditions of peak metamorphism for three aluminous (garnet–sillimanite) gneisses in the MnNCKFMASHTO chemical system using the internally consistent thermodynamic database (ds63) of Holland and Powell (2011) and the THERMOCALC (v. 3.45) software package (Powell and Holland 1988; Powell et al. 1998). The activity–composition models used in the calculations are from White et al. (2014a, 2014b) and include feldspar from Holland and Powell (2003), spinel–magnetite from White et al. (2002), and ilmenite–hematite from White et al. (2000). Phases modeled as pure end-members are quartz, rutile, aqueous fluid (H_2_O), kyanite, and sillimanite. Whole-rock analysis with titration provides the amounts of ferric and ferrous iron used in the modeling. No titration values are available for sample BC-16–10, where the molar amount of Fe^3+^ was set as 10% of the total iron, reflecting the reduced (e.g., graphite-bearing) mineral assemblage.

The phase assemblage in high-temperature metamorphic rocks is particularly sensitive to the amount of H_2_O in the modeled bulk composition (e.g., White et al. 2007), which may not be the same as the measured whole-rock sample due to post peak-metamorphic processes such as retrogression or weathering. In suprasolidus systems, the amount of H_2_O is usually adjusted so that the observed phase assemblage and mineral modes match those predicted in the modeling (Diener et al. 2008; Korhonen et al. 2010). Here, we follow the approach of Yakymchuk et al. (2015) by fixing pressure to 0.9 GPa (reasonable for the observed sillimanite-bearing assemblages) and adjusting H_2_O to the value where the solidus mineral assemblage and mineral proportions best match those in each sample. Although melt loss (c.f. Yakymchuk and Brown 2014) may have affected the rocks at Bissett Creek, the potentially residual compositions are more appropriate for modeling the peak P–T conditions—the goal of this modeling—than the protolith compositions (Johnson et al. 2021). The final modeled compositions are presented in Table 1.

**Table 1:** Whole-rock compositions used in phase equilibrium modeling

Sample	wt%												
	SiO_2_	TiO_2_	Al_2_O_3_	FeO	Fe_2_O_3_	MnO	MgO	CaO	Na_2_O	K_2_O	P_2_O_5_	LOI	Total
BC-16–10	82.49	0.43	8.17	n/a	2.80	0.03	1.14	0.19	0.29	3.04	0.07	0.66	98.56
CO-16–10	67.98	0.56	15.65	3.19	1.15	0.07	2.55	0.53	0.79	4.58	0.05	2.96	100.07
CO-16–05	69.53	0.63	14.65	3.50	1.50	0.07	2.26	0.39	0.79	5.89	0.04	0.39	99.64
		mol%											
Sample	Figure	H_2_O	SiO_2_	Al_2_O_3_	CaO	MgO	FeO	K_2_O	Na_2_O	TiO_2_	MnO	O	Total
BC-16–10	7a	1.04	86.85	5.07	0.22	1.79	2.22	2.04	0.30	0.34	0.03	0.11	100
CO-16–10	7b	9.91	68.27	9.26	0.57	3.82	3.55	2.93	0.77	0.43	0.06	0.43	100
CO-16–05	7c	2.03	74.35	9.23	0.45	3.60	4.34	4.02	0.82	0.51	0.07	0.60	100

*LOI* loss on ignition, *n/a* not analyzed

## Results

### Petrology and mineral compositions of graphitic gneisses

#### Quartzofeldspathic gneiss

**Fig. 4:**
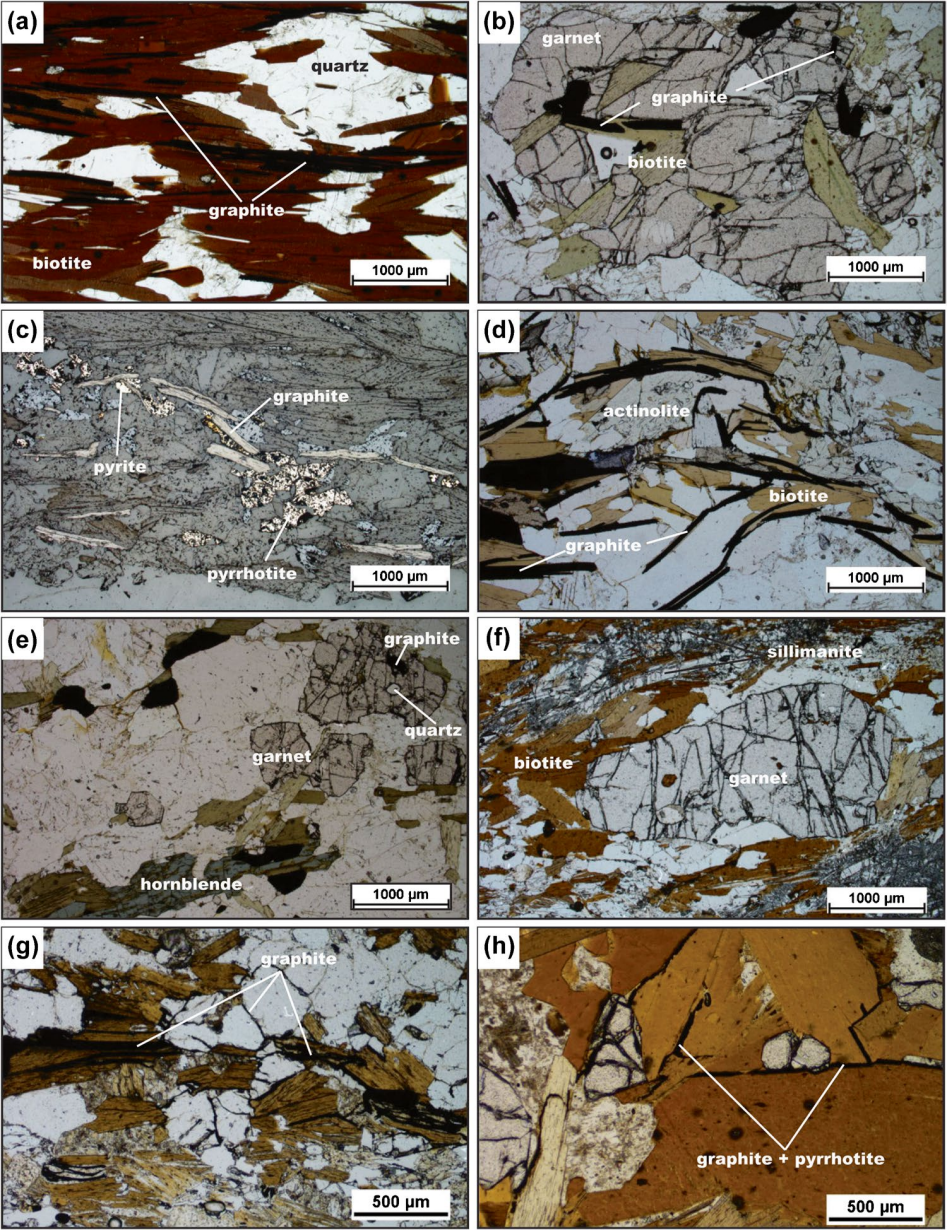
Thin section photomicrographs. All images in plane-polarized transmitted light except for (c), which is plane-polarized reflected light. **a** Interleaved biotite and graphite that define the foliation in graphite-bearing quartzofeldspathic gneiss. **b** Garnet with inclusions of graphite in graphite-bearing quartzofeldspathic gneiss. **c** Spatial association of pyrite and pyrrhotite with graphite in graphite-bearing quartzofeldspathic gneiss. **d** Flake graphite in clinopyroxene–quartzofeldspathic gneiss. Many graphite flakes are > 1 mm in length. **e** Graphite in garnet–hornblende gneiss. Garnet contains inclusions of graphite and quartz. **f** Melanocratic layer consisting of garnet + sillimanite + biotite in the graphite-bearing garnet–sillimanite gneiss. **g** Interleaved graphite and biotite in the garnet–sillimanite gneiss. Discontinuous graphite veinlets are present along quartz grain boundaries. **h** A veinlet of layered graphite and pyrrhotite along biotite grain boundaries in the garnet–sillimanite gneiss

Graphite-bearing quartzofeldspathic gneiss is the dominant graphite-bearing rock type at Bissett Creek. This unit is composed of quartz, plagioclase, and K-feldspar with variable amounts of biotite (5–25 vol.%) and minor amounts of graphite, pyrrhotite, and pyrite. Dispersed garnet and hornblende occur in some samples in minor proportions. Accessory minerals include titanite, allanite, chalcopyrite, and sphalerite. Biotite and graphite form a foliation (Fig. 4a), and this unit can contain a gneissosity defined by relatively biotite-rich domains and quartz-rich domains (Fig. 3e, 4a). Rare garnet contains inclusions of quartz, feldspar, biotite, and occasionally graphite and amphibole (Fig. 4b). Graphite is homogenously disseminated, comprising 5 to 10 vol.% of the unit. Graphite flake length varies from 0.1 to 6.0 mm and widths range from 0.05 to 0.3 mm; these are commonly interleaved with biotite and spatially associated with pyrrhotite and pyrite (Fig. 4c).

Garnet is almandine rich with *X*_Alm_ (molar Fe [Fe + Mg + Ca + Mn]) of ~ 0.5 and a notable amount of calcium, with grossular molar fractions of ~ 0.2; minor enrichment of Mn occurs at garnet rims but compositional zoning is otherwise flat. Plagioclase has *X*_An_ (molar Ca/[Ca + Na + K]) values of ~ 0.36–0.40 whereas K-feldspar has *X*_Or_ (molar K / [K + Na + Ca]) of 0.90–0.96 with up to ~ 2 wt.% Ba. Biotite has variable compositions with *X*_Fe_ (molar Fe/[Fe + Mg]) values of 0.26–0.44 (median 0.41). Matrix biotite has molar amounts of Ti (based on 22 oxygen) up to 0.24.

#### Clinopyroxene–quartzofeldspathic gneiss

This unit has the second-highest mode of graphite and is the only carbonate-bearing unit found at Bissett Creek. It contains a foliation defined by aligned biotite and graphite as well as centimeter to decimeter scale compositional layering into leucocratic—dominated by quartz, feldspar, clinopyroxene, and tremolite—and melanocratic domains that contain mostly biotite, clinopyroxene, and actinolite (Fig. 4d). Minor garnet, titanite, actinolite, calcite, pyrite, and pyrrhotite are also present. Rocks from this unit only show a single foliation, which is defined by a combination of the alignment of elongate and platy minerals as well as compositional layering. Graphite, pyrite, pyrrhotite, and other trace sulfides are disseminated throughout, although their modes are less than in the graphite-quartzofeldspathic gneiss. Clinopyroxene and calcite are xenoblastic; when present, calcite is in contact with graphite grains. Flake graphite (3 to 5 vol.%) ranges from 0.3 to 3.2 mm in length and from 0.05 to 0.12 mm in width. Graphite is commonly interleaved with biotite and spatially associated with biotite, diopside, tremolite, and actinolite (Fig. 4d) as well as pyrrhotite and pyrite. Mineral compositions were not determined for this unit based on the absence of useful assemblages for thermobarometry.

#### Garnet–hornblende gneiss

This unit is a minor component of the graphite-bearing rock types at Bissett Creek. The garnet–amphibole gneiss contains centimetric to decimetric leucocratic and melanocratic domains as well as a penetrative foliation defined by aligned biotite, hornblende, and graphite. Melanocratic domains are dominated by hornblende, garnet, biotite, and graphite (Fig. 4e) whereas leucocratic domains are dominantly composed of quartz, K-feldspar, and plagioclase. Accessory pyrrhotite, pyrite, and titanite also occur. Graphite is disseminated throughout the unit and is frequently found in close association with pyrrhotite and pyrite. Poikiloblastic garnet has unaligned inclusions of quartz, feldspar, biotite, hornblende, and graphite (Fig. 4e). Graphite flakes comprise 2 to 3 vol.% of the unit and range from 0.3 to 2.2 mm in length and from 0.05 to 0.15 mm in width. Graphite is commonly interleaved with biotite and spatially associated with biotite, hornblende, and garnet as well as pyrrhotite and pyrite.

Garnet exhibits minor compositional zoning but is almandine-rich (X_Alm_ ~ 0.50–0.58) with grossular molar fractions that vary from ~ 0.20 to 0.30. Hornblende has *X*_Fe_ values between 0.45 and 0.59 (median 0.50) and molar amounts of Si that vary from 6.7 to 6.3; these are variably classified as edenite, ferroedenite, pargasite, and ferropargasite according to the classification of Leake et al. (1997). Matrix plagioclase has *X*_An_ values ranging from 0.36 to 0.42 and one inclusion in garnet has a lower (*X*_An_ = 0.19) value. Most K-feldspars are *X*_Or_ of 0.85–0.95 with up to 1.2 wt% Ba. Biotite have *X*_Fe_ values ranging from 0.33 to 0.54 (median 0.48) with cations of Ti ranging from 0.02 to 0.24 (median 0.07).

#### Garnet–sillimanite gneiss

Garnet-sillimanite-biotite-graphite gneiss is rare at Bissett Creek. This unit contains a strong foliation defined by aligned sillimanite, biotite, and graphite and contains alternating leucocratic layers—dominated by quartz, K-feldspar, and plagioclase—and melanocratic layers dominated by garnet, biotite, sillimanite, and graphite (Fig. 4f, g). Poikiloblastic garnet is elongate in the foliation direction and contains inclusions of quartz and feldspar. Graphite and biotite are interleaved in the melanocratic layers. Graphite contents in this unit are relatively low (1–3 vol.%) and flakes vary in length from 0.2 to 1.4 mm and are from 0.05 to 0.1 mm wide, which is smaller than in other units. Graphite veinlets can be seen along grain boundaries (Fig. 4g) and are commonly associated with pyrrhotite and pyrite (Fig. 4h).

Garnet shows minor compositional zoning with highest Fe contents in the core that gradually decrease to the rims. *X*_Fe_ values are higher than in other units and vary from ~ 0.70 to 0.75 and grossular contents mostly ranging from ~ 0.05 to 0.10. Two alkali feldspars are present, with compositions of *X*_Or_ ~ 83–91 and *X*_Or_ ~ 30–39. Plagioclase is rare in the melanosome, and only one analysis was obtained and it is nearly pure albite. Biotite has *X*_Fe_ values between 0.74 and 0.44 (median 0.40) with moles of Ti ranging from 0.04 to 0.46.

### Carbon isotopes

**Fig. 5:**
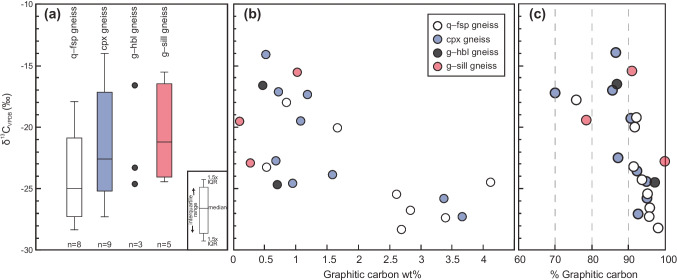
**a** δ^13^C values of whole-rock samples from different graphite-bearing units at Bissett Creek. For the box and whisker plots, the boxes extend to the interquartile range and the whiskers extend to furthest data point up to 1.5 times the interquartile range (IQR). **b** Variability of δ^13^C ratios with amount of graphitic carbon. δ^13^C values become lighter with more graphitic carbon. **c** Percentage of graphitic carbon in the sample from a total of graphitic and non-graphitic (e.g., carbonate) carbon in each whole-rock sample. Note that two samples from the garnet–sillimanite gneiss and one garnet–hornblende gneiss are not plotted on **b** and **c** because graphitic carbon contents were not measured.

The δ^13^C_VPDB_ values of samples from the Bissett Creek flake graphite deposit are summarized in Fig. 5a and reported in the electronic supplementary material (ESM Table S1). Carbon isotope values range from − 28.3 to − 14.0‰, with a median of − 23.3‰ (*n* = 24). Carbon isotope ratios from the quartzofeldspathic gneiss range from − 28.3 to − 17.9‰ with a median of − 24.9‰ (*n* = 8). Carbon isotope values from the clinopyroxene-gneiss range from − 27.2 to − 17.1‰ with a median of − 23.1‰ (*n* = 8). Three values from garnet–amphibole gneiss range from − 24.6 to − 16.6‰. Finally, δ^13^C values of the garnet–sillimanite gneiss vary from − 24.4 to − 15.5‰ (*n* = 5). These values are similar to an average value of − 21.2‰ (*n* = 2) of graphite concentrate from Bissett Creek (Taner et al. 2017). Carbon isotope ratios generally become lighter with increasing graphitic C content in samples (Fig. 5b).

Some samples contain trace amounts (< 1 vol.%) of carbonate minerals, and this influences the δ^13^C value of the measured whole-rock samples. Inferring the δ^13^C of the graphite from whole-rock analyses requires an assessment of a non-graphitic carbon component to the whole-rock δ^13^C values. The concentrations of graphitic carbon and total carbon are summarized in Table 2; the difference between these values is an approximation for the amount of non-graphitic carbon in the samples. Although there is a general relationship between heavier δ^13^C values and less graphitic carbon (compared with non-graphitic carbon), samples with > 90% graphitic carbon show a wide range of δ^13^C from − 28.3 to − 15.5‰ (Fig. 5c).

**Table 2:** Carbon contents of samples from Bissett Creek

Sample	Total C (wt%)	Graphitic C (wt%)	Difference (wt%)	Rock type
BC-16–11	4.38	4.11	0.27	Quartzofeldspathic gneiss
BC-16–19	1.12	0.85	0.27	Quartzofeldspathic gneiss
BC-16–28	0.59	0.54	0.05	Quartzofeldspathic gneiss
CO-16–01	2.74	2.69	0.05	Quartzofeldspathic gneiss
CO-16–02	2.95	2.83	0.12	Quartzofeldspathic gneiss
CO-16–03	3.55	3.40	0.15	Quartzofeldspathic gneiss
CO-16–04	1.82	1.67	0.15	Quartzofeldspathic gneiss
CO-16–07	2.74	2.61	0.13	Quartzofeldspathic gneiss
BC-16–06	3.96	3.67	0.29	Clinopyroxene gneiss
BC-16–07	1.19	1.08	0.11	Clinopyroxene gneiss
BC-16–08	1.72	1.59	0.13	Clinopyroxene gneiss
BC-16–13	1.01	0.96	0.05	Clinopyroxene gneiss
BC-16–14	0.84	0.72	0.12	Clinopyroxene gneiss
BC-16–17	1.70	1.19	0.51	Clinopyroxene gneiss
BC-16–22	3.54	3.37	0.17	Clinopyroxene gneiss
BC-16–38	0.78	0.68	0.10	Clinopyroxene gneiss
BC-16–20	0.54	0.47	0.07	Garnet–hornblende gneiss
BC-16–29	0.72	0.70	0.02	Garnet–hornblende gneiss
BC-16–25	0.26	0.28	-0.02	Garnet–sillimanite gneiss
CO-16–05	0.14	0.11	0.03	Garnet–sillimanite gneiss
CO-16–06	0.60	0.52	0.08	Garnet–sillimanite gneiss
CO-16–08	1.12	1.02	0.10	Garnet–sillimanite gneiss

### Sulfur isotopes

**Fig. 6:**
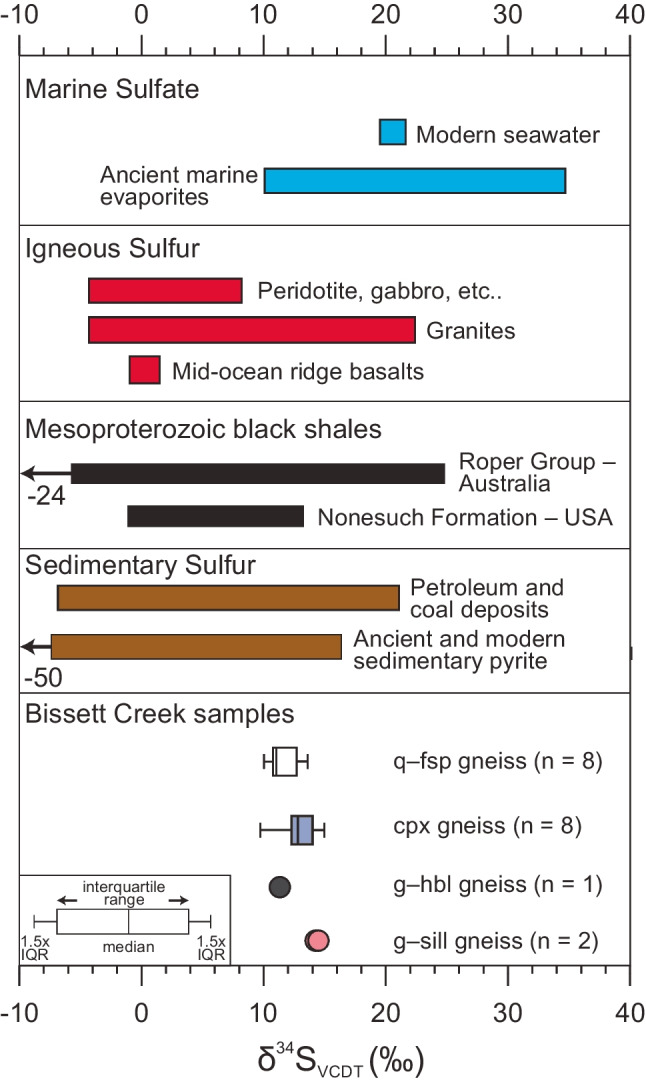
δ^34^S values of whole-rock samples from different graphite-bearing units at Bissett Creek compared with those from sulfur reservoirs (Nielsen 1979; Seal et al. 2000) and Mesoproterozoic black shales (Imbus et al. 1992; Johnston et al. 2008). For the box and whisker plots, the boxes extend to the interquartile range and the whiskers extend to furthest data point up to 1.5 times the interquartile range (IQR).

Whole-rock δ^34^S_VCDT_ results are summarized in Fig. 6 and reported in ESM Table S2. Nineteen samples from the Bissett Creek deposit yield δ^34^S_VCDT_ values ranging from 9.7 to 15.0‰, with a median of 12.7‰ (Fig. 6). Values of δ^34^S in the quartzofeldspathic gneiss ranged from 10.0 to 13.6‰, with a median of 11.0‰ (*n* = 8). Clinopyroxene gneiss yielded values ranging from 9.7 to 15.0‰, with a median of 12.8‰ (*n* = 8). One sample from the garnet–amphibole gneiss produced a value of 11.3‰. Two samples from the garnet–sillimanite gneiss yielded values of 14.2 and 14.3‰. There is no trend between δ^34^S values and total sulfur content or total carbon content.

### Thermobarometry

Garnet–biotite thermometry (Holdaway 2000) was applied to four garnet–sillimanite gneiss samples using the average matrix biotite and garnet core compositions, which are similar to garnet mantle compositions. Results range from ~ 620 to 720 °C (ESM Table S10). Considering the relatively high temperatures, it is possible that retrograde Fe–Mg exchange occurred during cooling, and these estimates are treated as minima (e.g., Pattison et al. 2003). The Ti-in-biotite thermometer (Henry et al. 2005) is less susceptible to retrograde cation exchange reactions, and we applied this to the same four samples. Two of these samples (CO-16–05 and BC-10–06) yield Ti-in-biotite temperatures of ~ 720 °C (*n* = 36) and ~ 725 °C (*n* = 16). One sample (CO-16–08) yielded a median Ti-in-biotite temperature of 580 °C (*n* = 8), and the last sample (CO-16–10) gave values < 600 °C (*n* = 4). We attribute some of this variability to uncertainty related to Ti-deprotonation exchange in systems with H_2_O–CO_2_ fluids (c.f. Henry et al. 2005). Furthermore, the paucity of ilmenite and the absence of rutile in our samples imply that these Ti-in-biotite estimates are minimal. Therefore, the minimum peak temperature for the Bissett Creek deposit based on garnet–biotite thermometry and Ti-in-biotite thermometry is 720 °C.

### Phase equilibrium modeling

**Fig. 7:**
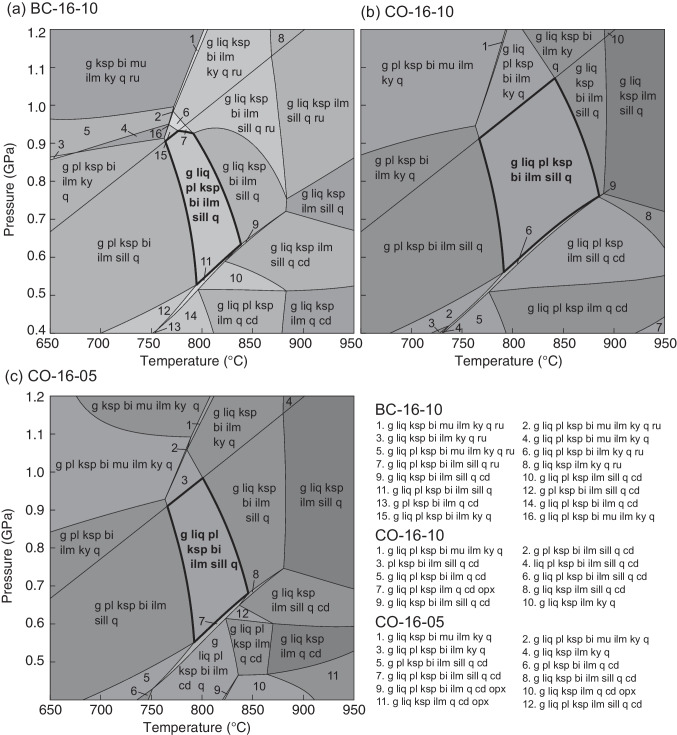
P–T phase diagrams for three garnet-sillimanite gneisses from the Bissett Creek deposit. The bold line outlines the P–T range of the field with the observed metamorphic mineral assemblage

Three garnet–sillimanite gneisses yield calculated phase diagrams with similar topologies. Each diagram contains an elevated solidus (~ 750–800 °C), a stability field of cordierite at low pressure and high temperature, and kyanite is stable at high pressure. These are all features common for phase equilibrium models of residual metapelites (e.g., Yakymchuk et al. 2017), and the results can be used to determine the peak P–T conditions (White et al. 2007). The inferred peak mineral assemblage from each sample from Bissett Creek contains garnet, sillimanite, biotite, quartz, K-feldspar, plagioclase, and ilmenite; the stability of this assemblage in the phase diagrams is ~ 0.5–1.0 GPa at 750–850 °C (Fig. 7). The upper temperature limit is constrained by the plagioclase-out boundary in each sample. Plagioclase is rare in our three samples and mostly found in the leucosome; if plagioclase is excluded from the peak assemblage, the estimated temperature is > 850 °C. However, the low modeled proportion of plagioclase above the solidus is considered within uncertainty of the modeling. The lower temperature limit is imposed by the elevated solidus temperature. The upper pressure limit is constrained by the absence of kyanite (and rutile) and the lower pressure limit by the absence of cordierite.

**Fig. 8:**
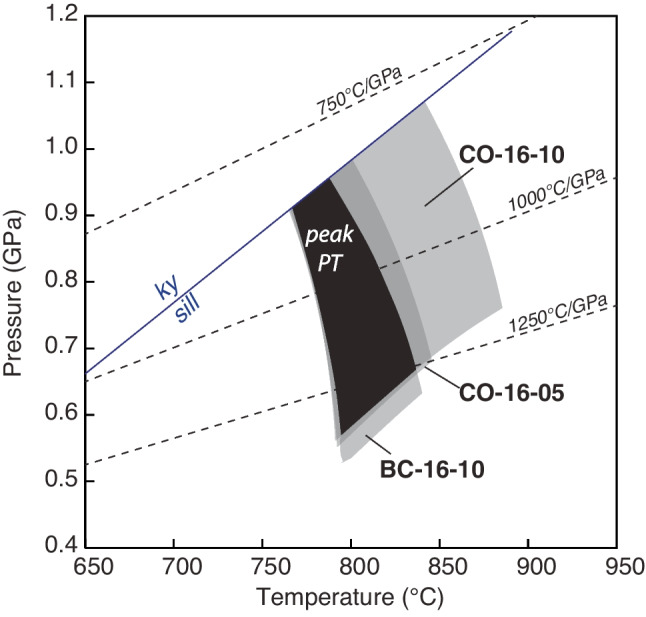
P–T pseudosection results for three garnet–sillimanite gneisses from Bissett Creek. The estimated peak P–T conditions are ~ 770–820 °C at ~ 0.6–0.9 GPa

Combining the modeled peak *P–T* stability fields for each sample, the best estimate for the peak *P–T* conditions at the Bissett Creek deposit is 0.55–0.95 GPa at 760–840 °C; this equates to an apparent thermal gradient between and 800 °C/GPa and 1250 °C/GPa (Fig. 8). These estimates are slightly higher than those from thermobarometry, and this is attributed to retrograde Fe–Mg exchange for the garnet–biotite thermometer and to the lack of a buffering Ti-bearing mineral (ilmenite, rutile) for the Ti-in-biotite thermometer. Consequently, our best estimate for the peak metamorphic temperatures for the Bissett Creek deposit is > 760 °C, similar to those calculated for rocks elsewhere in the Algonquin domain (Anovitz and Essene 1990; Timmermann et al. 2002; Kendrick and Jamieson 2016).

## Discussion

### Origin of carbon

**Fig. 9:**
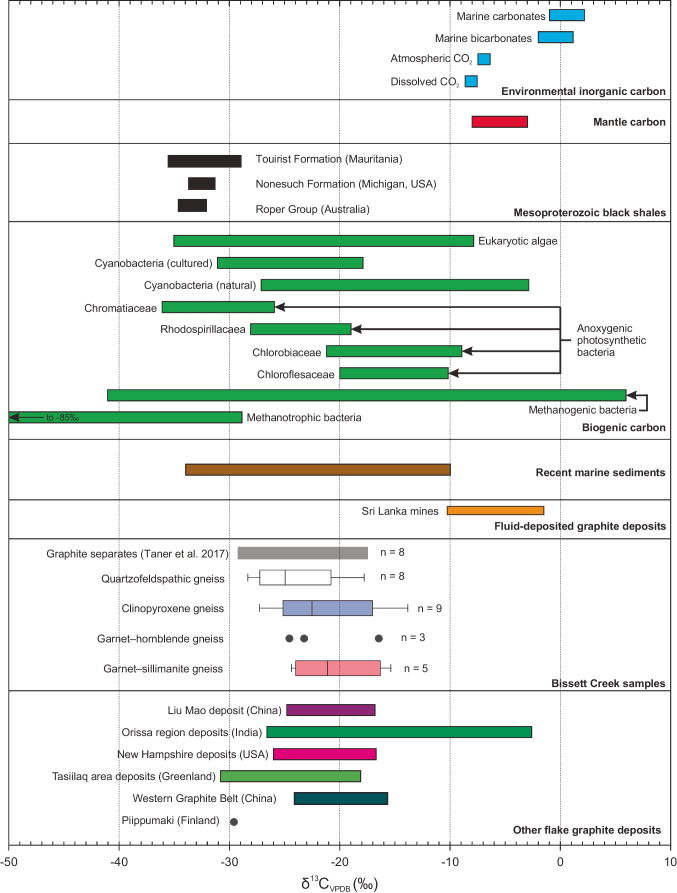
δ.^13^C values of whole-rock samples from different graphite-bearing units at Bissett Creek compared with potential carbon sources as well as values from other flake graphite deposits. Organic and marine carbon isotope values are from Schidlowski (1988) and Schidlowski (2001). Although the organic and marine carbon isotope values have large ranges, the values of organic carbon from minimally altered Proterozoic formations are expected to be only a few per mil (Strauss et al. 1992). Mantle carbonate values are from Javoy et al. (1986), Deines et al. (1991), and Viljoen (1995). Mesoproterozoic black shales are from Ho et al. (1990), Johnston et al. (2008), and Blumenberg et al. (2012). Carbon isotopes from graphite deposits are from Rankama (1948), Duke and Rumble (1986), Wilde et al. (1999), Volkert et al. (2000), Sanyal et al. (2009), Luque et al. (2012), Yang et al. (2014), Rosing-Schow et al. (2017)

Carbon isotope compositions of graphite are commonly used to infer the source of carbon involved in graphite formation (Wada et al. 1994; Luque et al. 1998, 2012; Wilde et al. 1999; Rosing-Schow et al. 2017; Parnell et al. 2021b). Biogenic carbonaceous materials generally have lighter isotopic compositions (δ^13^C of − 43 to − 3‰; Fig. 9) than carbon from the mantle (− 8 to − 3‰; Fig. 9) or from carbonates precipitated from seawater (~ 0‰) (Javoy et al. 1986; Schidlowski 1988, 2001; Deines et al. 1991; Deines 1992; Viljoen 1995). Most modern autotrophic organisms fall within the range of − 43 to − 3‰, except for methanogenic bacteria, whose carbon isotope ratios extend from − 41.5 to 6‰ and methanotrophic bacteria, which ranges from − 85.0 to − 29‰ (Fig. 9; Schidlowski 2001). The isotopic signature of organic carbon in the geologic record also varies with time; during Grenvillian orogenesis (ca. 1.1–0.95 Ga), δ^13^C_VPDB_ of organic carbon in the rock record ranges from approximately − 35.5 to − 12.5‰ with a mean of − 22‰ (Schidlowski 2001).

The carbon isotope compositions of whole-rock samples from Bissett Creek—thought to be equivalent to graphite given the large proportion of graphitic carbon in the samples—range from − 28.3 to − 14.0‰. Although rare, some samples contain very minor amounts of calcite, and its contribution to the carbon budget of individual samples is indicated by differences in the measured amounts of total carbon and graphitic carbon (Table 2). Samples with lower fractions of graphitic carbon (~ 70–90%) yield the heaviest δ^13^C values whereas the samples with the lowest δ^13^C values contain > 90% graphitic carbon (Table 2; Fig. 5c). This suggests that some of the relatively high δ^13^C values may record a mixed signature of graphite and calcite from samples. However, samples with > 90% graphitic carbon (Table 2) have δ^13^C values that range from − 28.3 to − 15.5‰ (Fig. 5c), which are similar to previous values of − 29.1 to − 15.6‰ from graphite separates from Bissett Creek (Taner et al. 2017; Garland 1987). Therefore, we consider the ~ 14‰ spread in δ^13^C values to reflect that of graphite from the Bissett Creek deposit.

Graphite deposits generated exclusively from the graphitization of organic matter are expected to have a narrow range of carbon isotope values because the precursor material is similar across the area of mineralization and then undergoes the same metamorphic conditions (Luque et al. 2012). For example, minimally altered Proterozoic kerogens usually have a range of values of < 5‰ across individual formations (Strauss et al. 1992). The range of values at Bissett Creek is ~ 14‰ and is inconsistent with metamorphism of organic material with a homogeneous composition. This continuum of isotopic compositions from Bissett Creek deposit could have been developed as a result of three factors. The isotopic variability of carbon at the Bissett Creek flake graphite deposit could be in response to (1) metamorphism, (2) exchange with isotopically heavier minerals (i.e., carbonates), and (3) isotope fractionation during precipitation of graphite from a hydrothermal fluid or anatectic melt.

High-grade metamorphism can shift the isotopic signature of carbon in graphite to heavier values with increasing temperature (Hoefs and Frey 1976; Wada et al. 1994). From amphibolite- to granulite-facies, this shift is estimated to be ~ 5‰ (Barker and Friedman 1969; Wada et al. 1994) and is probably caused by the release of isotopically light methane during metamorphism (Morikiyo 1986; Luque et al. 2012). Even considering this shift to heavier values, the δ^13^C values at Bissett Creek are still within the range of biogenic carbon. Additionally, the graphite-bearing units likely experienced similar metamorphic conditions which would result in a narrower range of δ^13^C values.

Another mechanism that can shift the δ^13^C signature of graphite from that of the original carbon source is isotope exchange between graphite and carbonates during metamorphism (Hahn-Weinheimer and Hirner 1981; Dunn and Valley 1992). Isotopic fractionation between graphite and calcite is smaller at higher temperature (Dunn and Valley 1992). At the estimated peak metamorphic temperatures of ~ 750–800 ºC at Bissett Creek, the difference between calcite and graphite is ~ 2–3‰. However, calcite–graphite carbon isotope fractionation is larger at lower temperatures, such as during slow retrograde cooling. This cooling could facilitate further exchange and cause larger ranges of carbon isotope composition in the calcite and graphite (Dunn and Valley 1992). Although calcite is mostly absent in rocks at Bissett Creek, the clinopyroxene-quartzofeldspathic gneiss contains rare calcite, and many of the Ca-rich minerals (clinopyroxene, tremolite, actinolite) may be relics of decarbonation reactions (e.g., Valley and Essene 1980). We speculate that some of the variation of δ^13^C values of graphite at Bissett Creek may have been caused, in part, by calcite–graphite isotopic exchange during prograde metamorphism when decarbonation reactions are most likely to occur. However, the variability of δ^13^C in the calcite-bearing rocks (clinopyroxene–quartzofeldspathic gneiss) and in the calcite-absent rocks (graphite-bearing quartzofeldspathic gneiss) indicates that calcite–graphite exchange is not the primary process causing the range of δ^13^C values at Bissett Creek.

A potential carbon-rich protolith for some of the rocks at Bissett Creek are Mesoproterozoic black shales; these rocks have generally comparable carbon and sulfur isotope ratios with those at Bissett Creek (Fig. 9). In general, the carbon isotope compositions of whole-rock samples and graphite separates (Taner et al. 2017) from Bissett Creek are isotopically heavier than those from Mesoproterozoic black shales. This could indicate modification during metamorphism (up to 5‰ heavier; Barker and Friedman 1969; Wada et al. 1994), exchange with isotopically heavy carbonate minerals (2 to 3‰; e.g., Dunn and Valley 1992), or may suggest that the potential precursor shale to the paragneiss was isotopically heavier than the others.

Although carbon isotopic variability of graphite from Bissett Creek could be related to fractionation during metamorphism and calcite–graphite exchange, a third possible mechanism is hydrothermal precipitation of graphite from carbonic fluids. Hydrothermal graphite deposits have δ^13^C signatures that range from − 33.7 to − 1.6‰ because of fluid–rock interactions and carbon isotope fractionation during graphite formation (Luque et al. 2012). Single or multi-component Rayleigh fractionation (Ray 2009) between CO_2_ and CH_4_ fluid from a single homogenous isotopic source with a δ^13^C of − 26‰ can produce a spread of values from − 33 to − 10‰ (Taner et al. 2017). However, to explain this range in the multi-component system, a starting fluid composition of dominantly CH_4_ (not CO_2_) must be present. Considering that CH_4_ is a minor fraction of metamorphic fluids in graphite-bearing systems at the high temperatures (e.g., Chu and Ague 2013; van Hinsberg et al. 2021; Yakymchuk et al. 2021), it is unlikely that a multi-component system was responsible for fractionation. Instead, a single component fractionation model can account for the spread of δ^13^C values and is consistent with the observation that rocks with the highest carbon content have the lowest δ^13^C values (Fig. 5b).

Although metamorphic fractionation and graphite–calcite exchange could have influenced the C isotope ratios of graphite at Bissett Creek, isotopic fractionation during graphite precipitation from a carbonic fluid (or melt) generated during metamorphism is probably the dominant cause of δ^13^C variability. The presence of grain-boundary graphite between quartz (microveinlets in Fig. 4g, h) is compatible with—although not diagnostic of—graphite precipitation from a fluid/melt (e.g., Galvez et al. 2013; Rumble 2014). In addition, similar features to the graphite/sulfide bands in the Bissett Creek samples (Fig. 4h) have been associated with sulfide anatexis (Mishra and Bernhardt 2009). The petrographic association of graphite and sulfide minerals at Bissett Creek (Fig. 4c, h) is also consistent with fluid-deposited graphite (Bernard and Papineau 2014). Combining the measured spread in δ^13^C values with these petrographic observations from Bissett Creek, we suggest that the large to extra-large flake size of graphite at Bissett Creek may represent a hybrid metamorphic–hydrothermal origin where some in situ biogenic carbon was mobilized and re-precipitated within the deposit during metamorphism.

### Petrogenesis

**Fig. 10:**
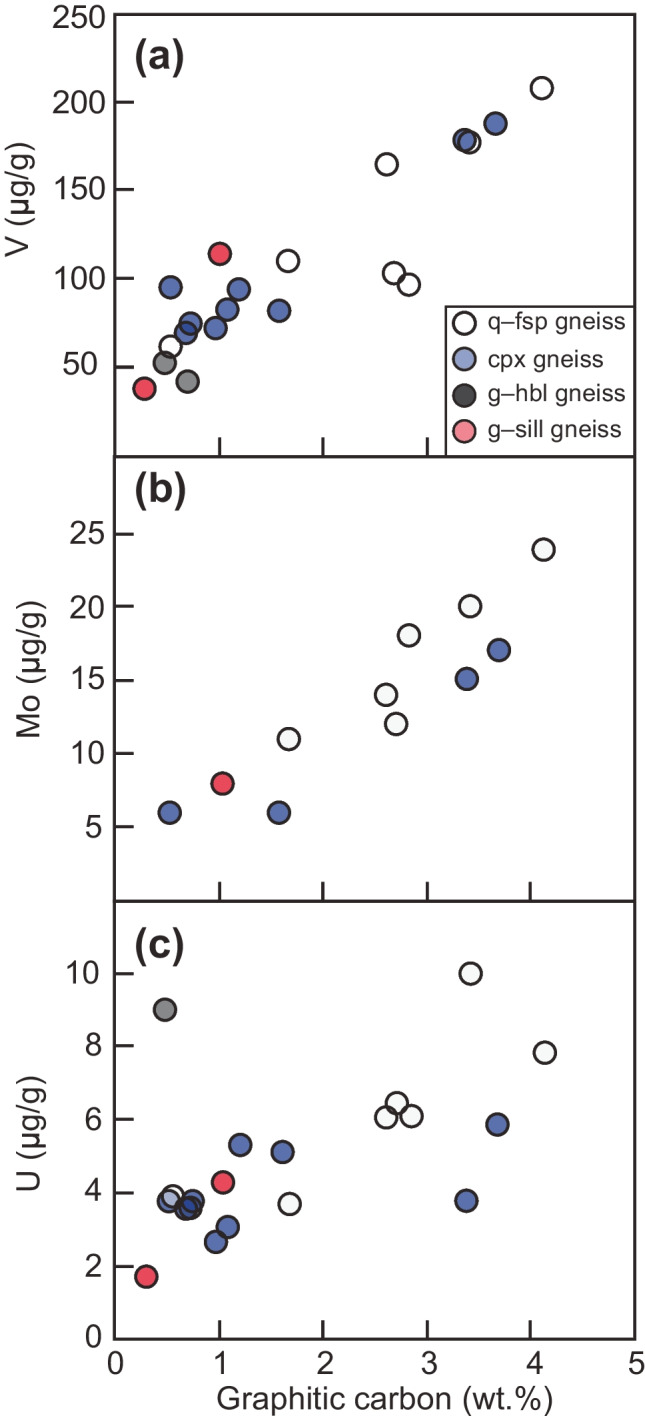
Concentrations of redox sensitive elements (V, Mo, U) in whole-rock graphite-bearing samples from Bissett Creek. For the trace elements, some values below the limit of detection are not plotted.

A hybrid metamorphic–hydrothermal genetic model for flake graphite at Bissett Creek is compatible with the observed textures and carbon isotope signatures. Hydrothermal fluids were likely generated in situ, resulting from metamorphic dehydration and decarbonation reactions, and perhaps further influenced by partial melting. The sharp boundary between the mineralized graphitic gneisses (Fig. 3b) and the unmineralized barren gneisses support in situ fluid development. In other deposits, such as in the Tasiilaq area of Greenland, hydrothermal fluids are interpreted to be externally derived, and mineralization cross-cuts all units regardless of protolith (Rosing-Schow et al. 2017). As organic material represents the main source of carbon at Bissett Creek (Fig. 9), it is likely that bacterial organic matter was deposited into a sedimentary sequence containing arenitic, psammitic, and pelagic rocks, with some calcareous layers represented now by the clinopyroxene-gneiss, consistent with either a shallow marine or closed basin environments (e.g., Volkert et al. 2000). The deposition of organic carbon in conjunction with sedimentation processes is supported by the positive correlations between graphitic carbon and redox-sensitive metals such as V, Mo, and U (Fig. 10; similar to black shales) and with sulfur isotope (Fig. 6) and carbon isotope compositions (Fig. 9) (Nielsen 1979; Schidlowski 1988, 2001).

Covariation between graphitic carbon content and redox-sensitive metals (V, Mo, and U) (Fig. 10) coupled with S and C isotope compositions (Figs. 6 and 9) suggests that the inferred protolith of the aluminous rocks at Bissett Creek was likely deposited under anaerobic conditions, similar to Mesoproterozoic black shales. During burial and heating associated with Grenvillian orogenesis (Ketchum and Davidson 2000; Rivers et al. 2012), carbon-rich residues of complex organic compounds became gradually dehydrated during diagenesis and metamorphism leading to the development of graphitic carbon and later graphite (e.g., Buseck and Beyssac 2014). Primary formation of graphite through the graphitization process at Bissett Creek is supported by the disseminated nature of the graphite mineralization, the interleaving of graphite with metamorphic minerals, and that flake graphite is the dominant crystal habit in most units; vein graphite is absent except for rare microscopic veinlets (Fig. 4g, h). Metamorphism to upper amphibolite conditions caused incipient partial melting. If graphite was an unreactive restitic mineral, then local melt extraction may also locally increase its proportion in residual graphitic gneisses. However, partial melting may have also remobilized carbon from graphite into a silicate melt, or into an immiscible carbonic fluid (c.f. Chu and Ague 2013). Peak *P–T* estimates from this study and from nearby regions (Anovitz and Essene 1990; Timmermann et al. 2002; Kendrick and Jamieson 2016) suggest that temperatures were sufficient to induce partial melting, whereas small graphite microveinlets (Fig. 3g, h) provide petrographic evidence of graphite remobilization. The spread of δ^13^C values compatible with a single component fluid fractionation model at Bissett Creek (Taner et al. 2017) also supports a metamorphic hydrothermal component of graphite mineralization. This hypothesis could be further tested with in situ δ^13^C analysis across individual graphite grains (e.g., Farquhar et al. 1999; Satish-Kumar et al. 2011). Nevertheless, high-temperature metamorphism and partial melting may have been a key factor for carbon mobilization and the development of large- to extra-large flake graphite at Bissett Creek.

### Exploration for large-flake graphite deposits

There are several deposits with large to extra-large flake graphite around the world that are potential analogues for graphite mineralization at Bissett Creek. There are several key features that are common and provide important exploration criteria (Table 3). We now describe three general observations that characterize deposits of large to extra-large flake graphite worldwide.

**Table 3:** Properties of international flake graphite deposits

Deposit	Ore style	T (°C)	Max flake size (mm)	δ^13^C (‰, VPDB)	References
Bissett Creek, Canada	Flake	650–750	6	− 31.7 to − 14.0 (*n* = 30)	Garland (1987); Taner et al. (2017)
Liu Mao, China	Flake	500–850	Nd	− 24.8 to − 16.8 (*n* = 22)	Wilde et al. (1999)
Orissa Region, India	Flake/vein	620–945	Nd	Flake: − 26.6 to − 2.4 (*n* = 14); Vein: − 24.4 to − 8.8 (*n*-16)	Sanyal et al. (2009)
Spaulding Suite, USA	Flake/Vein	600–750	1.0	− 26.0 to − 16.7 (*n* = 39)	Duke and Rumble (1986)
New Jersey Highlands, USA	Flake	680–760	1.0–2.0	− 28.4 to − 16.4 (*n* = 16)	Volkert et al. (2000)
Tasiilaq Area, Greenland	Flake	600	5.0	− 30.8 to − 18.1 (*n* = 24)	Rosing-Schow et al. (2017)
Western Graphite Belt, China	Flake/Vein	900	3.0	− 24.1 to − 15.8	Wang (1989); Yang et al. (2014); Zhang et al. (2014); Zhong et al. (2019)
Northern Norway	Flake	810–835	3.5	− 18.6 to − 22.5	Gautneb et al. (2020); Engvik et al. (2021); Parnell et al. (2021a)
Rautalampi and Käpysuo, Finland	Flake	470–590	1.6	Nd	Al-ani et al. (2020)
Piippumaki, Finland	Flake	740	1.0	− 29.6 (*n* = 1)	Palosaari et al. (2020); Rankama (1948)

*Nd* no data

First, the host rocks of each deposit are inferred to have a clastic sedimentary protolith—these vary from sandstones to greywackes to mudstones—with or without a carbonate sedimentary component. Organic-rich facies in sedimentary sequences are commonly associated with enrichment of redox-sensitive elements (e.g., U, V in black shales) and sulfur; Wilde et al. (1999) inferred that the formation of graphite in the Liu Mao graphite deposit was attributed to the metamorphism of U- and V-rich black shales under upper amphibolite to granulite-facies *P–T* conditions. At the Bissett Creek deposit, positive correlations between Mo, V, and possibly U with graphitic carbon (Fig. 10) suggest that organic-rich black shale enriched in the redox-sensitive metals is a reasonable protolith for some of the rocks. In addition, flake graphite deposits worldwide commonly have associated sulfides (e.g., pyrite and pyrrhotite), which have δ^34^S values compatible with those found in black shales (Fig. 6).

A second observation is that flake graphite deposits commonly yield carbon isotope signatures consistent with organic materials (Table 3; Fig. 9); this contrasts with vein graphite deposits that can have graphite with higher δ^13^C values that reflect carbonate and the mantle carbon input (Luque et al. 2012). This also supports the postulate that the graphite mineralization system formed in a (meta)sedimentary package that included organic-rich facies.

Third, flake graphite deposits form during upper amphibolite to granulite facies with and without accompanying anatexis. Although the specific role of anatexis in graphite deposits is not clear, melt extraction from a graphite bearing migmatite has the potential to increase the concentration of graphite. Because of the limited solubility of CO_2_ in silica-rich melts (i.e., granitoids) at crustal temperatures and pressures (Holloway et al. 1992; Blank et al. 1993), and because of mass balance, the extracted melt will leave behind a more C-rich bulk residual composition (Chu and Ague 2013). This may explain the variable concentrations of graphitic carbon in the same geological units from Bissett Creek (Fig. 5b). However, anatexis may also play a role in the remobilization of graphite (Wilde et al. 1999); we speculate that this is a possible reason for the relatively large flake size of graphite at Bissett Creek compared with other flake graphite deposits worldwide (Table 3).

In subsolidus systems, late fluids can remobilize graphite to form new morphologies. For example, in the Orissa region, graphite mineralization occurred during in situ metamorphism of organic material with subsequent remobilization and vein mineralization related to igneous intrusions (Sanyal et al. 2009). These graphite-rich veins are located along the intrusion margins and within intrusion-related shear zones. Similarly, flake graphite mineralization in the Tasiilaq Area was originally the result of graphitization, but was remobilized during metamorphism and deposited from hydrothermal fluids; this is partly based on the observation that disseminated flake graphite cross-cuts a number of lithological boundaries (Rosing-Schow et al. 2017). In the Spaulding Suite graphite occurrences, disseminated flake graphite—originally formed from metamorphism of in situ organic matter—is found in metasedimentary wall rocks as well as the intrusive granitoid plutons. The disseminated flake graphite in the plutons is thought to represent xenocrystic material that was assimilated into the igneous rocks during intrusion. Assimilation of this sedimentary material into the magma caused the formation of veinlets and spherules during graphite remobilization as a carbon-bearing fluid during intrusion (Duke and Rumble 1986). This is supported by similar δ^13^C values of graphite in the metasedimentary rocks and in the intrusive granitoids (Duke and Rumble 1986). Lastly, the Xinghe Deposit in China contains strata-bound flake graphite mineralization and graphite veins; these veins are interpreted to be derived from fluids formed from high-grade metamorphism (Yang et al. 2014).

Considering the common observations from flake graphite deposits worldwide, the key exploration indicators are clastic sedimentary sequences with organic-rich protoliths that experienced upper-amphibolite to granulite-facies metamorphism. Although in situ mineralization of organic-rich material is the primary cause of flake graphite mineralization, both subsolidus and suprasolidus processes may refine graphite deposits by remobilizing carbon and enhancing graphite grain size. Further research can explore the potential role of carbon mobilization in melt-rich (Chu and Ague 2013) and fluid-rich (Stewart and Ague 2018; Evans and Tomkins 2020) metamorphic systems and its implications for the increasing the grain size of flake graphite.

## Conclusions

Large- to extra-large flake graphite at the Bissett Creek in the southeastern Grenville Province of Canada formed during high-temperature moderate-pressure (> 760 °C and 0.5 to 0.9 GPa) metamorphism of a predominately clastic sedimentary sequence. Graphite flakes (up to ~ 6 mm in size) are predominantly disseminated in various rock types, but also occurs as microveinlets at grain boundaries—both morphologies of graphite are commonly associated with sulfide minerals. Whole-rock carbon isotope compositions (− 28 to − 14‰, δ^13^C_VPDB_) suggest that graphitic carbon has a biogenic origin. However, the wide range of values suggest hydrothermal (or anatectic) mobilization of carbon and precipitation of graphite. In situ metamorphism of organic matter coupled with graphite coarsening via CO_2_-bearing fluid (or melt) may be important for forming deposits with extra-large flake graphite.

## Supplementary Information

Below is the link to the electronic supplementary material.

Supplementary file1 (PDF 102 KB)

Supplementary file2 (XLSX 187 KB)

## Data Availability

All data used in this study are in the supplementary material.
