# Supplementary material for: Petrogenesis of extra-large flake graphite at the Bissett Creek deposit, Canada
Source: Miner Depos. 2022 Dec 5;58(4):731–50. doi: 10.1007/s00126-022-01145-9 (PMC9992087; doi:10.1007/s00126-022-01145-9)
Supplement: Supplementary file 1 — Supplementary file1 (PDF 102 KB) [file 126_2022_1145_MOESM1_ESM.pdf]

Supplementary material for:

Petrogenesis of an extra-large flake graphite deposit, Bissett Creek, Canada

Cameron Drever, Carson Kinney, Chris Yakymchuk

Analytical Methods

Whole-rock geochemistry

Major elements and trace elements were analyzed as part of the Actlabs 4 Lithoresearch package. Major element concentrations were determined by Inductively Coupled Plasma Optical Emission Spectrometer (ICP-OES), while trace element concentrations were determined using an Inductively Coupled Plasma Mass Spectrometer (ICP-MS). To prepare the samples for analysis, the material is melted using lithium metaborate and lithium tetraborate as fluxes in an induction furnace. The molten material is then poured directly into a solution of 5% nitric acid, which is stirred until the molten material is completely dissolved. The final solution is used in the ICP-OES and ICP-MS for analysis.

Total carbon and sulfur concentrations are measured using Actlabs package 4F on an infrared (IR) detector. To start the procedure, an accelerator material is combined with 0.2g of sample material. The combined material is then placed in an induction furnace and heated until the carbon and sulfur combust. The combustion takes place in a pure oxygen environment, causing the formation of CO, CO_2, and SO_2. The CO is changed into CO_2 in a catalytic heater assembly. These gases then flow into the IR cell where they absorb specific wavelengths of the infrared spectrum. The absorption of the wavelengths reduces the amount of energy that reaches the IR detectors, which can be used to determine the total amount of CO_2 or SO_2 gas passing by and correspondingly the total amounts of carbon and sulfur.

Graphitic carbon is measured in a very similar manner to total carbon and sulfur (part of package 4F), but there are two main differences. The first difference is that 0.5g of sample material is used per analysis. The second is that the sample undergoes a multistage furnace treatment to remove all other forms of carbon prior to analysis, leaving only carbon in the form of graphitic carbon behind. Afterwards, the sample is subjected to the same process outlined above for combustion and detection.

Carbon isotopes

Samples were measured from the same whole-rock powders used for geochemistry (section 4.1). Samples for carbon isotope analysis were sent to the Queen's Facility for Isotope Research (QFIR) and samples were processed in compliance with their standard carbon isotope analysis procedures. 0.2-0.6 grams of each sample is weighed into tin capsules. Combustion was followed by analysis in a Costech ECS 4010 Elemental Analyzer coupled to a Thermo-Finnigan DeltaPlus XP Continuous-Flow Isotope Ratio Mass Spectrometer (CF-IRMS). The $\delta^{13}C$ values obtained from the measurement are reported using delta (δ) notation with units of permil (‰) relative to VPDB international standard. Precision of the VPDB standard was 0.2‰. The quality assurance and control program at QFIR includes the analysis of: (1) Certified reference materials and secondary standards, (2) random duplication of unknown samples and (3) blanks. These

materials constitute 10% of the total analyses when the instrument is on-line and 20% when the instrument is off-line. The primary reference material was the VPDB.

Sulfur isotopes

Sample material for sulfur isotope analysis was taken from the same whole rock powders used for geochemistry (section 5.0). Samples were sent to the Environmental Isotope Laboratory (UW-EIL) at the University of Waterloo. Samples ranging from 1.3 mg to 3.1 mg (depending on the concentration of sulfur) were loaded in 3.5 mm x 5mm tin capsules. Loaded tin capsules are dropped into the Costech ECS 4010 Elemental Analyzer coupled to an Isochrom CF-IRMS which measures the isotopic composition of the sulfur present. The isotopic composition of the sample is determined through combustion conversion (1000ºC reactor, 90ºC column, 115ml/min. He) of the solid sulfur-bearing materials into SO_2 gas. This gas is then run through a Costech Instruments Elemental Analyzer connected to an Isochrom Continuous Flow Isotope Ratio Mass Spectrometer. The $\delta^{34}S$ values are reported using the delta (δ) notation with units of permil (‰) relative to the VCDT international standard with a precision of 0.3‰. The UW-EIL quality assurance and quality control program consists of two aspects. Firstly, during an analytical run, at least 20% of all materials analyzed are either in-house references or international standards. For sulfur isotopes, the international standards used are: IAEA-SO-5, IAEA-SO-6, NBS-127 ($BaSO_4$), NBS-123 (ZnS), IAEA-S1, IAEA-S2, IAEA-S3 (AgS). Secondly, to ensure repeatability, 1 in 10 analyses are duplicated.

Mineral compositions

Mineral compositions were determined quantitatively for garnet, biotite, amphibole, plagioclase, potassium feldspar and sulfides using a JEOL JXA-8530F field-emission electron microprobe at the Earth and Planetary Materials Analysis (EPMA) Lab at Western University (Canada). Garnet was analyzed using 1–25 1 μm spots along a line to assess compositional variation with an accelerating voltage of 15 kV and 20 nA current. Biotite, amphibole, plagioclase, potassium feldspar and sulfides were analyzed using 5 μm spots (two on each mineral, one on the rim and one in the core) with the same accelerating voltage and current. Acquisition time was ~3 minutes. Calibration of the instrument was undertaken prior to the start of analyses using a combination of natural and synthetic standards. The data was reduced using the built-in ZAF corrections in the JEOL software.

Supplementary Data tables

Table S1. Carbon isotope values
Table S2. Sulfur isotope results
Table S3. Lithogeochemistry
Table S4. Sample locations
Table S5. Biotite Compositions
Table S6. Plagioclase Compositions
Table S7. Alkali feldspar compositions
Table S8. Garnet Compositions
Table S9. Amphibole Compositions
Table S10. Results of Garnet–biotite thermometry (Holdaway, 2000)
Table S11. Results of Ti in biotite thermometry (Henry et al., 2005)
